# Complexity data science: A spin-off from digital twins

**DOI:** 10.1093/pnasnexus/pgae456

**Published:** 2024-11-12

**Authors:** Frank Emmert-Streib, Hocine Cherifi, Kimmo Kaski, Stuart Kauffman, Olli Yli-Harja

**Affiliations:** Predictive Society and Data Analytics Lab, Faculty of Information Technology and Communication Sciences, Tampere University, Korkeakoulunkatu 7, 33720 Tampere, Finland; Laboratoire Interdisciplinaire Carnot de Bourgogne, Université de Bourgogne, 9 Avenue Alain Savary, BP 47870, 21078 Dijon Cedex, France; Department of Computer Science, Aalto University, P.O. Box 11000, FI-00076 Espoo, Finland; The Alan Turing Institute, British Library, 96 Euston Rd, London NW1 2DB, United Kingdom; Institute for Systems Biology, 401 Terry Ave N, Seattle, WA 98195, USA; Predictive Society and Data Analytics Lab, Faculty of Information Technology and Communication Sciences, Tampere University, Korkeakoulunkatu 7, 33720 Tampere, Finland; Institute for Systems Biology, 401 Terry Ave N, Seattle, WA 98195, USA

**Keywords:** digital twin, data science, complexity science, simulation, learning

## Abstract

Digital twins offer a new and exciting framework that has recently attracted significant interest in fields such as oncology, immunology, and cardiology. The basic idea of a digital twin is to combine simulation and learning to create a virtual model of a physical object. In this paper, we explore how the concept of digital twins can be generalized into a broader, overarching field. From a theoretical standpoint, this generalization is achieved by recognizing that the duality of a digital twin fundamentally connects complexity science with data science, leading to the emergence of complexity data science as a synthesis of the two. We examine the broader implications of this field, including its historical roots, challenges, and opportunities.

## Introduction

Complex systems have been studied for decades, but an essay by Warren Weaver (1) from 1948 is often credited with outlining a vision for the field, which he summarized as the study of “organized complexity.” Complex systems denote particular systems or phenomena characterized by a multitude of interconnected elements or agents that interact, leading to the emergence of distinctive properties and behaviors. A prominent example is Boolean networks, which were introduced as a simplified model for gene regulation (2). However, many complex systems have been studied across multiple domains, including ecosystems, human behavior, and economic markets (3–6).

In recent years, complex systems have once again come into the center of interest, particularly as part of digital twin research. While the concept of a digital twin originated in manufacturing (7), it has only recently been proposed for other areas including medicine, health, and epidemiology. Importantly, these domains have a long history of complex systems research preceding the advent of digital twins (8–11).

In this paper, we extend the concept of a digital twin. First, we demonstrate that, from a theoretical perspective, a digital twin integrates concepts from complexity science and data science. Second, we argue that a digital twin is merely one instance of the broader potential enabled by this integration. To highlight the broader possibilities, we introduce the term “complexity data science,” denoting a new field, and discuss its various implications. Third, from the newly gained perspective of complexity data science, we look retrospectively back and discuss similarities to complex adaptive systems (CAS).

## Digital twins

The underlying idea behind a digital twin is often stated in simplified form as follows (12): a digital twin is an ideal concept a digital representation of a real-world object that closely mirrors its physical counterpart. This digital representation takes the form of a computer-based simulation model, while the real-world object can encompass systems or processes with a physical presence, such as an engine, biological cell, or economic process. The first applications of this concept can be found in manufacturing and engineering (7, 13, 14).

While digital twins originated in manufacturing, the concept has recently gained significant interest in various scientific fields, including health-related concerns, climate change mitigation, urban development, economic regulation, and sustainability (15–17). Although the benefits of digital twins in these newer fields are yet to be fully explored, the concept is generally met with high expectations.

From a theoretical perspective, a digital twin combines two key features that set it apart from other approaches: simulation and learning (18). In the following sections, we discuss both of these components in detail.

### Simulation

For obtaining a digital representation of a real-word object, simulations are needed to establish mathematical models. These simulation models emulate crucial aspects of the physical twin, capturing relevant dynamic behavior under different conditions. This necessitates a functional description in a holistic rather than a reductionistic manner, often involving different forms of complex networks.

A scientometric analysis of published articles on digital twin research in Emmert-Streib et al. (19) demonstrated that there are 2 distinct groups of application fields, with the onset of the former preceding the latter by several years. The first group comprises applications in manufacturing and engineering, while the second group includes fields such as medicine, immunology, and epidemiology. Regarding the establishment of simulation models, it is important to note that digital twin simulation models for manufacturing and engineering are based on descriptions of the mechanical problem and are often related to physical theory. In contrast, our understanding of problems in biology, medicine, and related fields is limited compared with physical theory. Therefore, approximation models based on dynamical and complex systems are used in these fields, providing simplified descriptions that are still useful for applications. Despite the large number of different approaches, ranging from ordinary differential equations to agent-based models, their commonality is to provide mechanistic models.

Although simulations and modeling of biological or epidemiological problems have been used before the advent of digital twins, the next feature discussed in the following section sets digital twins apart.

### Learning

A fundamental issue with any simulation model is to ensure its correctness, which requires knowledge of the structure and parameters of the model. Traditionally, the structure and parameters are derived from experimental observations, and once determined, a fixed model is used. This places a huge burden on this step as everything that follows depends on its accuracy.

Digital twins work differently. Instead of using a one-step process, as described previously, they employ a continuous learning schema within which their parameters or structure are iteratively updated over time. This requires new data from the physical object to be available continuously or frequently, allowing the digital twin model to be continuously calibrated. As a result, the quality of a digital twin may be poor initially, but it can improve significantly as more data become available over time.

Together, the combination of simulation and learning distinguishes digital twins from ordinary simulation models, explaining the tremendous interest in digital twins across scientific fields.

### Exploration of “what-if” scenarios

**Fig. 1.**
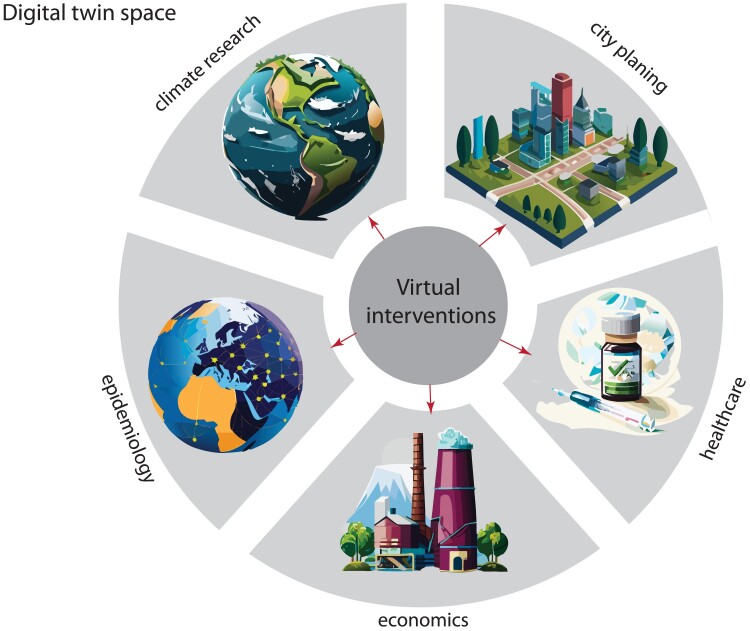
Digital twins allow the study of “what-if” scenarios corresponding to virtual interventions of the underlying physical twin. Shown are 5 application domains in digital twin space: climate research, city planning, healthcare, economics, and epidemiology.

A consequence of the mechanistic nature of digital twins is that their models allow the exploration of “what-if” scenarios. This means that digital twins enable to examine the ramifications of virtual interventions performed on the structure of a model. Consequently, this offers the ability to gain insights into the effects of interventions. Examples of such interventions and their effects on problems could correspond to the following (see Fig. 1):

- Climate research: transitioning to alternative energy sources and the effect on climate and CO_2_ emissions
- Epidemiology: effect of lockdown and social distancing on the epidemic spread of a virus
- Economics: change in taxation on the gross domestic product of a country.
- Medicine: change of medications and the effect on the well-being of patients
- Urban development: influence of city planning on traffic flow and traffic accidents

It is important to highlight that these interventions are “virtual” because they are only performed on the model, that is the digital twin, and not in the real world. This underscores one of the key benefits of digital twins compared with conventional prediction models, which do not allow such interventions. In addition, this approach is resource-efficient, enabling cost- and time-efficient investigation of the effects of interventions.

On a related note, we would like to highlight that “what-if” scenarios are similar to counterfactual explanations, a topic of growing interest within the explainable artificial intelligence (AI) community (20, 21). Specifically, counterfactual explanations help us to understand “what could have happened if the input to a model had been altered in a specific way” (22). This approach is crucial for developing reliable machine learning models, especially in applications where decisions need to be explained, such as in healthcare, law, finance, or medicine. A notable example of counterfactual explanations in biomedicine can be found in Metsch et al. (23).

## Complexity data science

So far, we have focused closely on digital twins and two of their key functionalities, namely simulation and learning. Now, we move beyond the boundaries set by this framework. By adopting a theoretical perspective, we can see that the complex systems underlying simulations are connected to complexity science (24), while learning is deeply rooted in data science (25).

Complexity science is a broad multidisciplinary field that explores complex phenomena in various domains, including physics, biology, sociology, economics, and more (24, 26). Its primary focus is to unravel the fundamental principles, recurring patterns, and emergent behaviors inherent in complex systems. Complexity science strives to formulate overarching theories and frameworks that can be applied to a wide spectrum of complex systems, irrespective of their specific characteristics or domains. Since their inception, complexity science and the concept of complexity have defied a straightforward definition. Instead, they are better understood by identifying common themes, underlying threads, and the tools used in their study (27, 28). It should be noted that this multidisciplinary field draws on a range of mathematical approaches, including stochastic processes, information theory, network theory, and statistical physics, illustrating the diverse and multifaceted nature of the discipline.

In contrast, data science is a field focused on extracting knowledge and insights from data using various techniques from statistics, machine learning, and AI (29). It involves the collection, processing, and analysis of large sets of data to identify patterns, make predictions, and inform decision making. Data science leverages machine learning algorithms, statistical models, and computational tools to handle complex data and derive actionable insights, focusing on practical applications and solutions to real-world problems across diverse areas.

The combination of simulation and learning inherent of digital twins has significant potential that extends beyond traditional digital twins. This integration could include specialized variations or extensions of digital twins that, while not strictly adhering to their foundational principles, still effectively merge simulation and learning. Fundamentally, the fusion of complexity science and data science can be termed “complexity data science,” representing the rise of a new field.

In the following, we discuss two possible applications of complexity data science that are not directly related to digital twins.

### Explainable AI

Deep learning methods based on neural networks with many hidden layers are currently among the best and most widely used techniques for numerous prediction tasks, such as classification, regression, and image processing (30–33). However, deep neural networks have been criticized for their opaqueness, as these models lack interpretability. For this reason, such approaches are called black-box models (34). This is a particular problem in application domains like medicine and healthcare, in which understanding abstract predictions and decisions is desirable and necessary due to policy regulations (35, 36). Commonly, this issue is summarized under the term “explainable AI” (37, 38).

**Fig. 2.**
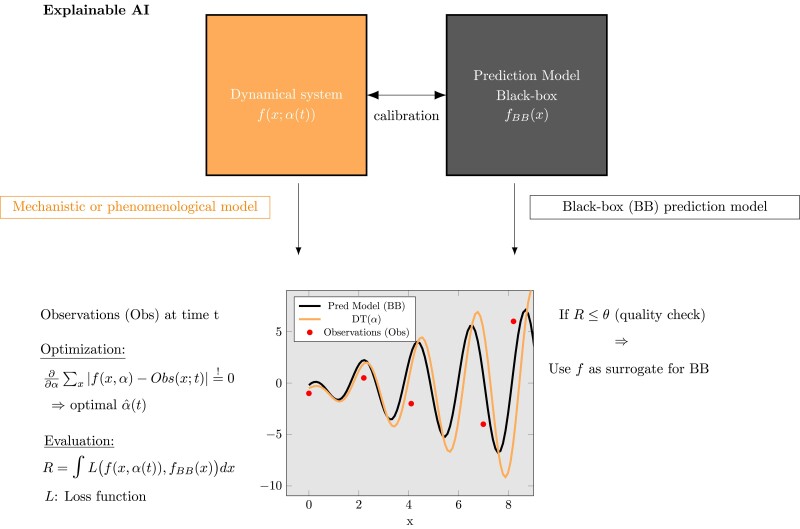
Explainable artificial intelligence (AI). By calibrating a mechanistic or phenomenological model to a black-box prediction model, one can utilize such a model as surrogate for gaining interpretable insights into the underlying processes, while retaining the predictive power of the original black-box model.

While complexity data science cannot directly convert deep neural networks into explainable models, it can offer a systematic approach to circumvent this problem. By employing a dynamical system as a mechanistic or phenomenological model, one can calibrate it to align with a deep neural network for a specific task (see Fig. 2). This involves adjusting the parameters of the dynamical system through an optimization mechanism to match the predictions of the deep neural network. Practically, this could be achieved with physics-informed neural networks (39, 40), which estimate such parameters using deep learning with modified cost functions that allow for consideration of constraints set by the dynamical system itself. The quality of this calibration can be evaluated using task-specific error scores. For example, for a regression task, scores such as mean squared error or *R*^2^ (coefficient of determination) can be used, and for a classification task, accuracy or the F1 score can be applied.

After determining the calibration score, it can be evaluated to see if the accuracy of the calibrated model is sufficient considering task-specific criteria. If it is, then the mechanistic model can serve as a surrogate for the prediction behavior of the deep neural network, with the added benefit of being interpretable. Depending on the characteristics of the data and the relationships among the features, the quality of the model's interpretations can range from associations to causal relations. It is clear that causal relations are most desirable; however, even association networks underlying such models may hold value by providing a broad view of intricate dynamical behavior, especially, in case of very complex problems. Examples for the utility of association networks can be found in studies about gene regulations (41) and investigations of the organizational structure of the brain (42).

Leveraging interpretable models derived from complexity science allows us to move away from the opaque black-box models commonly found in data science, such as deep neural networks, toward more explainable solutions. This example illustrates how a complexity data science approach can enhance conventional data science methods by addressing the challenges associated with explainable AI.

### Online complex systems

In data science, the form of learning where new data becomes available in a sequential order and is used to continuously improve a prediction model is commonly referred to as online learning or incremental learning (43). Although this is not the most prevalent form of learning due to the requirement of needing additional data obtained over time, there are situations where such data are available. Examples are healthcare monitoring, annual gross domestic products of countries, or stock price predictions.

For complex systems, the inclusion of new data obtained sequentially can imply time-dependent parameters or a changing structure, as new data may lead to updating these parameters. Agent-based models are generally useful for modeling complex systems with time-dependent parameters and evolving network structures, especially when individual heterogeneity and adaptive behaviors are important. It is crucial to emphasize that the time scale of the sequentially observed data is within the life cycle of the modeled entity, such as a patient or cell, rather than spanning evolutionary time scales over many generations. This means one can use general optimization mechanisms for parameter updates, as these do not need to be consistent with evolutionary mechanisms, such as simulating mutations. Hence, general learning algorithms from machine learning or statistics can be utilized. This leads to online complex systems with more versatile capabilities than traditional complex systems for modeling non-stationary and nonlinear processes.

It is important to note that for stationary processes, online, complex systems should improve, as more data means larger sample sizes available for learning. This enables increased flexibility to allow for the eventual convergence of the model toward the desired solution. As a side note, this touches upon philosophical considerations in science, particularly the challenge of induction dating back to David Hume (44).

### Complexity of deep learning

Another application of complexity data science could be in enhancing our understanding of learning in deep neural networks. In this context, learning refers to the optimization of parameters according to specific rules. Interestingly, despite being biologically inspired, learning in neural networks is usually not conducted via neurobiological Hebbian learning, but rather through variations of the mathematical stochastic gradient descent. Still, there is a great potential to expand our understanding of deep learning by method transfer from other areas.

For instance, complexity measures based on entropy and information can provide insights into the working mechanisms of deep learning. A particular approach is provided by the information bottleneck principle (45, 46). The information bottleneck framework allows studying the trade-off between preserving relevant information and compressing or discarding irrelevant information during learning. This makes the information flow of the learning dynamics throughout the different layers of a deep neural network quantifiable. Consequently, this could be utilized to improve parameter optimization itself, thereby enhancing prediction capabilities.

## Historic traces

At this point, we would like to revisit previous findings or instances resembling complexity data science principles or applications. An example of this is Vemuri's classic book, *Modeling of Complex Systems: An Introduction*, published in 1978 (47). Although the primary use of complex systems is the qualitative description of dynamical behavior, this book contains a chapter titled “Forecasting,” which provides an in-depth discussion and presentation of statistical analysis methods. This is remarkable because it underscores Vemuri's early recognition of the significance of forecasting, or prediction, in conjunction with complex systems.

Another connection to an early study from 1986 can be found in Farmer et al. (48). This work modeled the immune system simulation as a dynamical system based on coupled differential equations. Importantly, the model utilizes a learning mechanism via genetic operations that functions on a time scale of days, not generations. Notably, the similarity between this model and classifier systems—a general-purpose method for analyzing data—introduced by Holland (49) was highlighted and comparatively discussed. Recognition of such a connection is important as the communities investigating adaptation in natural and learning in artificial systems are largely separated.

The simulation model of the immune system and similar approaches are widely referred to as CAS, which dynamically respond and adapt to environmental changes (50). Although there is no universally accepted formal definition of a CAS, they are commonly described as systems exhibiting emergent behavior, adaptability, and self-organization (51). In the last 30 years, CAS has been extensively studied in biology, ecology, and immunology, indicating their relevance to natural systems. To a lesser extent, human behavior has also been studied, with notable applications in economics (52, 53).

It is important to emphasize that CAS provide primarily a qualitative understanding, rather than detailed quantitative predictions. This distinguishes them from digital twins and complexity data science because accurate numerical predictions are essential to make a real-world impact. This includes also the quantitative evaluation and the testing of predictions to judge the accuracy and quality of such models.

## Challenges and opportunities

The capabilities of complexity data science are domain independent; however, different applications tend to favor specific model types. For example, models in systems biology are largely based on differential equations (54), while agent-based approaches keep gaining traction in economic models (55). The differing characteristics of these models may affect their calibration, such as finding explainable models or estimating parameters. Therefore, these differences need to be systematically studied through subsequent case studies to develop recommendations for applications and optimal experimental design.

**Fig. 3.**
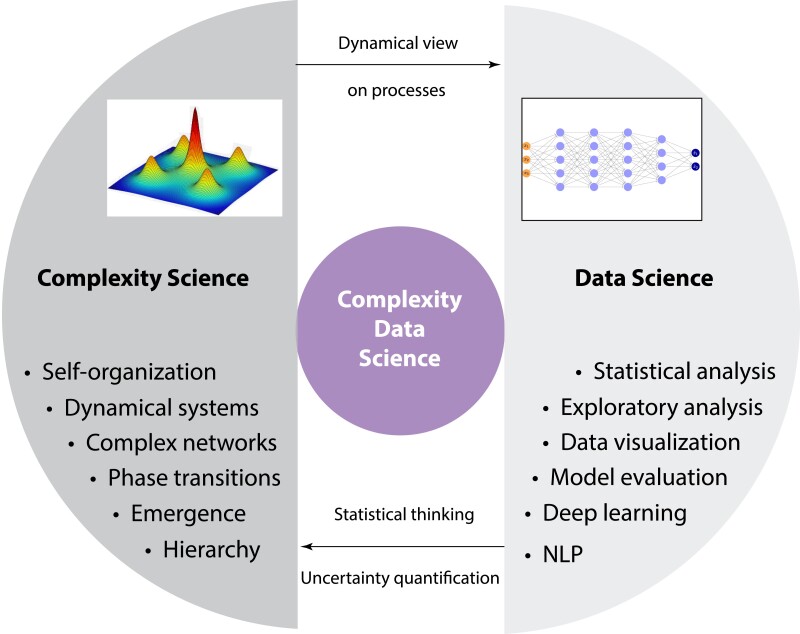
Education in complexity data science involves integrating the complementary fields of complexity science and data science. NLP = natural language processing.

Another challenge is the education of future researchers. For this an integrated curriculum for complexity data science is needed (see Fig. 3). Currently, degree programs in data science, machine learning, AI, and statistics are mostly within computer science departments. Meanwhile, programs in complexity science and complex systems are typically found in physics or applied mathematics departments. This means that there is a well-established infrastructure providing the necessary education for a professional career for the two branches of complexity data science. Unfortunately, the overlap among those branches is severely underdeveloped, which can lead to a variety of deficits. Succinctly, the most significant ones are the following:

- Deficits in data science education: lack of a dynamic view on processes
- Deficits in complexity science education: lack of statistical thinking

While data science highlights the importance of data, it typically overlooks the processes responsible for generating that data. For instance, data generation is often oversimplified, portrayed merely as drawing samples from a distribution. However, this simplistic view lacks the depth provided by understanding systems being governed by differential equations or agent-based modeling, which offer mechanistic or phenomenological insights. Such approaches provide a dynamic perspective on natural, social, or technological processes underlying the data. Additionally, the concepts like emergence, hierarchy, or self-organization necessitate this dynamic view for comprehensive understanding.

The complexity science often neglects the statistical complexities and uncertainties inherent in the systems it studies. While it provides valuable insights into emergent phenomena and system dynamics, it does not fully account for real-world data's statistical variability and uncertainties. By “lack of statistical thinking,” we mean a deficiency in understanding quantitative approaches for quantifying uncertainties in numerical predictions. This includes measurement errors in data and uncertainties in estimated parameters.

Overcoming these academic silos is not straightforward because education is, to this day, department based, and a transdisciplinary degree program faces administrative challenges. Hopefully, the prospects of the added value provided by complexity data science are recognized to establish such a much needed program.

## Conclusion

In our increasingly complex world, we must constantly enhance our problem-solving tools to efficiently address emerging challenges. The present moment calls for expanding our capabilities through integrating complexity science and data science into a field we call complexity data science. This interdisciplinary approach holds the potential to provide novel insights and capabilities, pushing the boundaries of what can be achieved in domains that traditionally focus on either simulation or learning.

## Data Availability

No data were used.
